# The Underlying Mechanism of 3-Hydroxyphthalic Anhydride-Modified Bovine Beta-Lactoglobulin to Block Human Papillomavirus Entry Into the Host Cell

**DOI:** 10.3389/fmicb.2019.02188

**Published:** 2019-09-26

**Authors:** Chen Hua, Yun Zhu, Congquan Wu, Lulu Si, Qian Wang, Long Sui, Shibo Jiang

**Affiliations:** ^1^Key Laboratory of Medical Molecular Virology (MOE/NHC/CAMS), School of Basic Medical Sciences, Fudan-Jinbo Functional Protein Joint Research Center, Fudan University, Shanghai, China; ^2^National Laboratory of Biomacromolecules, Institute of Biophysics, Chinese Academy of Sciences, Beijing, China; ^3^Medical Center for Diagnosis and Treatment of Cervical Disease, Obstetrics and Gynecology Hospital, Fudan University, Shanghai, China

**Keywords:** 3HP-β-LG, human papillomavirus, virus entry inhibition, L1 protein, mechanism of action

## Abstract

We have previously demonstrated that 3-hydroxyphthalic anhydride (3HP)-modified bovine beta-lactoglobulin (3HP-β-LG) is highly effective in inhibiting entry of pseudovirus (PsV) of high- and low-risk human papillomavirus (HPV) into the target cell. Intravaginally applied 3HP-β-LG-containing vaginal gel could significantly inhibit HPV infection and reduce viral load in the cervical region. However, we still do not understand the underlying molecular mechanism by which 3HP-β-LG is able to inhibit HPV infection. Here, though, we showed that 3HP-β-LG did not inactivate HPV PsV, but rather blocked entry of HPV PsV into the target cell *via* its interaction with virus, not cell. It bound to the positively charged region in the HPV L1 protein, suggesting that 3HP-β-LG binds to HPV L1 protein through the interaction between the negatively charged region in 3HP-β-LG and the positively charged region in HPV L1 protein, thus competitively blocking the binding of HPV to the receptor on the basement membrane in vaginal mucosa. Although 3HP-modified chicken ovalbumin (3HP-OVA) also carries high net negative charges, it exhibited no anti-HPV activity, suggesting that the interaction between 3HP-modified protein and HPV L1 protein relies on both electrostatic and matchable conformation of the binding sites in both proteins. When topically applied, 3HP-β-LG did not enter the host cell or blood circulation. These findings suggest that 3HP-β-LG targets HPV L1 protein and blocks HPV entry into the host cell, thus being safe and effective for topical application in the treatment of HPV infection.

## Introduction

Cervical cancer is the second most common cancer among women in China (Chen et al., 2016). Almost all these cases result from the persistent infection of human papillomavirus (HPV) (Bosch et al., 2002). HPVs are a large viral family consisting of about 200 different types (Bernard et al., 2010). Among them, 18 types have high oncogenic properties, and these are regarded as the high-risk types, such as HPV16, HPV18, and HPV58. The high-risk types of HPV have relatively low self-clearance rate compared to the low-risk types of HPV (Safaeian et al., 2008). To prevent the infection of these high-risk HPVs and the occurrence of cervical cancer, several prophylactic vaccines, like Cervarix, Gardasil, and Gardasil 9 (Deschuyteneer et al., 2010; McCormack, 2014), have been developed and approved for marketing in many countries. However, no therapeutic vaccines or drugs have been approved for clinical use to treat HPV-infected patients.

HPV is a non-enveloped, double-stranded DNA virus. Its capsid is made up of two proteins, major protein L1 and minor protein L2. They are both involved in receptor binding and viral entry. Analysis of the atomic structure of native *T* = 7 HPV virus-like particle (VLP) reveals that 72 L1 pentamers form the icosahedral shell of HPV (Baker et al., 1991; Li et al., 2016). The L1 pentamer strongly binds to HPV receptors, such as heparan sulfate proteoglycans (HSPGs), on the basement membrane to mediate viral entry and infection (Kines et al., 2009). The highly potent neutralizing antibodies elicited by HPV vaccines were found to bind to the L1 protein and prevent HPV infection (Li et al., 2018). Thus, the L1 pentamer is regarded as the most important target for development of antiviral agents against HPV infection.

Viral entry inhibitors represent a class of antiviral agents that inhibit virus infection by blocking virus entry into the host cells. Jiang et al. reported the first peptide-based HIV entry inhibitor, SJ-2176, in 1993 (Jiang et al., 1993) and the first HIV entry inhibitor-based anti-HIV drug, enfuvirtide, was approved by U.S. FDA for clinical use in 2003 (Dando and Perry, 2003). Later, Jiang and colleagues have reported that anhydride-modified proteins such as 3-hydroxyphthalic anhydride (3HP)-modified proteins are potent virus entry inhibitors against a number of enveloped viruses, such as HIV (Neurath et al., 1996), herpes simplex virus (HSV) (Neurath et al., 1996), and Ebola virus (EBOV) (Li et al., 2017). In 2012, Jiang’s group reported that 3HP-modified bovine beta-lactoglobulin (3HP-β-LG) could also inhibit entry into the target cell of the pseudovirus (PsV) of non-enveloped virus, HPV (high-risk HPV16 and HPV18, and low-risk HPV6) (Lu et al., 2013). In the clinical trial, the topical application of vaginal gel containing 3HP-β-LG was proven to be very safe and highly effective in suppressing HPV infection and reducing viral load in vaginal mucosa (Guo et al., 2016a,b). However, we still do not understand the molecular mechanism by which 3HP-β-LG inhibits HPV infection. In this study, we found that while 3HP-β-LG could not inactivate HPV PsV, it was effective in inhibiting HPV PsV entry into the host cell *via* its interaction with virus, not the cell. Unlike β-LG, 3HP-β-LG could bind with L1 protein and the peptides derived from the positively charged region in the HPV L1 protein, suggesting that 3HP-β-LG inhibits HPV infection by binding, *via* its negatively charged region, to L1 protein, and thus competitively blocking HPV binding to the receptor on the basement membrane in vaginal mucosa, which then inhibits HPV from entering and replicating in the host cell. We have also shown that topically applied 3HP-β-LG does not enter the host cell and blood circulation. Therefore, the 3HP-β-LG-containing formulations can be safely used in vagina for treatment of HPV infection and prevention of cervical cancer development.

## Materials and Methods

### Reagents

3-Hydroxyphthalic anhydride (HP), 2,4,6-trinitrobenzenesulfonic acid (TNBS), human serum albumin (HSA), β-lactoglobulin (β-LG), chicken ovalbumin (OVA), and bovine serum albumin (BSA) were purchased from Sigma. Horseradish peroxidase (HRP)-conjugated goat anti-human IgA and IgE antibodies were purchased from Abcam (UK). HRP-conjugated rabbit anti-mouse IgG antibody was purchased from Dako (Denmark). The HPV16- and HPV-58-L1/L2-expressing plasmids and Luciferase pCLucf plasmid were kindly provided by Dr. John Schiller at the Laboratory of Cellular Oncology, National Cancer Institute, NIH, MD, USA.

### Chemical Modification and Characterization of Anhydride-Modified Proteins

Anhydride-modified proteins were produced as previously described (Lu et al., 2013). Briefly, each kind of protein was dissolved with 0.1 M phosphate buffer (pH 8.5) at a final concentration of 20 mg/ml. Afterward, protein solutions were mixed with anhydrides (HP at 1 M in DMSO) to a final concentration of 60 mM by the addition of five equal aliquots at 20-min intervals, while the pH was adjusted to 9.0 with 4 M NaOH after each mixing. All mixtures were kept at 25°C for two more hours and then dialyzed against PBS.

### 3-Hydroxyphthalic Anhydride Modified Bovine Beta-Lactoglobulin-Mediated Inhibition Against Human Papillomavirus Pseudovirus Entry Into HeLa or HaCaT Cells

The HPV pseudovirus was produced as previously described^1^. Briefly, 293 T cells, which had been seeded in a 10-cm culture dish (4 × 10^6^ cells) at 16 h before transfection, were transfected with a mixture of HPV16-L1/L2-expressing plasmid (p16sheLL) and pCLucf plasmid for HPV16 PsV, or a mixture of HPV58-L1/L2-expressing plasmid (p58sheLL) and pCLucf plasmid for HPV58 PsV, using VigoFect (Vigorous Biotechnology Corp.). The cells were suspended in 0.5 ml of lysis buffer and incubated for 24 h at 37°C with slow rotation. The lysate was cooled on ice for 5 min, mixed with 5 M NaCl solutions to adjust the concentration of NaCl to 0.85 M, and further kept on ice for 10 min. Then, the lysate was centrifuged at 5,000 × *g* for 10 min at 4°C, and quantified for the concentration of L1/L2 capsid protein levels. To remove aggregates, the pseudovirion stock was filtered before use for the neutralization assay. The inhibitory activity of 3HP-β-LG against HPV PsV entry into HeLa or HaCaT cells was detected as previously described (Surviladze et al., 2012; Kwak et al., 2014). Briefly, HeLa or HecCatT cells were seeded at 1.5 × 10^4^ cells in 100 μl of 10% FBS DMEM (DMEM-10) per well in a 96-well plate, followed by a culture at 37°C overnight. 3HP-β-LG or β-LG was serially diluted in DMEM and incubated with 100 μl of HPV PsV (equal to 35 ng L1). Mixtures were added to HeLa or HaCaT cells and incubated at 37°C for 16 h. After replacement of the culture supernatants with free medium and culture for additional 72 h, cells were lysed for measurement of luciferase activity, according to the manufacturer’s manual (Promega, Madison, WI, USA).

### Enzyme-Linked Immunosorbent Assay

3HP-β-LG diluted in PBS was coated onto all wells of a 96-well polystyrene plate (Corning, USA) at 4°C overnight. After blocking with protein-free blocking buffer (Thermo Fisher Scientific, USA), HPV L1 (type16 or 18) protein at 1 μg/ml was added into the wells for 50 μl/well, and the plate was incubated at 37°C for 1 h. After washing with PBST three times, mouse anti-HPV L1 (type16 or 18) antibody (Abcam, UK) was added at a 1: 1,000 dilution. After incubation at 37°C for 1 h and washing again, HRP-conjugated rabbit anti-mouse IgG antibody (Dako, Denmark) was added for 1 h. After washing and adding tetramethylbenzidine (Sigma, USA), the absorbance was measured at 450 nm. Similar protocols were used for detection of 3HP-β-LG in the serum of rhesus monkeys and 3HP-β-LG-specific IgA and IgE in the vaginal swab eluates, which were leftover samples from the clinical trials for evaluating the *in vivo* safety and efficacy of the intravaginally applied 3HP-β-LG-containing vaginal gels (Registry No.: ChiCTR-TRC-12002016) (Guo et al., 2016a,b).

### Isothermal Titration Calorimetry for Measurement of the Binding Ability of 3-Hydroxyphthalic Anhydride Modified Bovine Beta-Lactoglobulin and Beta-Lactoglobulin to Peptides

Two positively charged peptides of HPV L1 protein, L1-I (residues 474–488: LKAKPKFTLGKRKAT) and L1-II (residues 493–505: STSTTAKRKKRKL), were synthesized by a standard solid-phase Fmoc method as previously reported (He et al., 2007). The peptide solution (1 mM) was prepared in 50 mM phosphate buffer (pH 7.4) and added into the titration sample cells. 3HP-β-LG and β-LG were also dissolved in phosphate buffer to 50 μM and then added to the titration needle. Isothermal titration calorimetry (ITC) analysis was carried out at constant temperature of 18°C with stirring speed of 1,000 rev/min. Each titration volume was 2 μl, and the titration interval was 60 s for the first drop and 120 s for the others.

### Time-of-Addition Assay

HeLa cells were plated in a 96-well plate (for 1.0 × 10^5^/well) overnight. Then, cells were incubated with 3HP-β-LG at a final concentration of 15 μg/ml at 0.5 h before or 0, 0.5, 1, 2, 4, 8, 12, 24, and 48 h after addition of HPV PsV (equal to 36 ng L1), followed by an incubation at 37°C for 16 h. After replacement of culture supernatant with fresh medium and an incubation for additional 72 h, cells were lysed to determine the entry inhibition ratio, according to the manufacturer’s manual (Promega).

### Cell Wash Assay

HeLa cells in 96-well plates were preincubated with 100 μl of 3HP-β-LG (5 μM) or β-LG (5 μM) at 37°C for 1 h and then washed with DMEM three times before addition of HPV PsV. For the control, 3HP-β-LG- or β-LG-pretreated HeLa cells were not washed before HPV PsV was added. HPV PsV entry into HeLa cells was detected as described above.

### Temperature Shift Assay

The plated HeLa cells and 3HP-β-LG mixture were both prechilled at 4°C for 30 min. Then, the cells were incubated with the 3HP-β-LG mixture at 4°C for 12 h. After washing with cold PBS buffer twice, the cells were further incubated at 37°C for 72 h. Then the cells were lysed to determine luciferase activity, according to the luciferase assay system manual (Promega, USA).

### Experiment on Rhesus Macaques

The experiment on rhesus macaques was conducted under ethical guidelines and approved by the Ethics Committee of the Institute of Laboratory Animal Science, Chinese Academy of Medical Sciences and Peking Union Medical College (approval number: ILAS-VL-2015-001). In brief, the rhesus macaques were randomly divided into treatment group and control group. In the treatment group, vaginal gel containing 3HP-β-LG (1.8 mg per dose) was dispersed into carbomer gel and administered intravaginally or rectally. In the control group, only carbomer gel was applied. Then, 2 ml of blood was collected before or after injection at −1 h, 0 h, 30 min, 1 h, 2 h, 3 h, 6 h, 12 h, and 24 h. The serum was separated after condensation.

### Immunofluorescence Staining

To determine whether 3HP-β-LG could enter into HeLa or 293 T cells, cells (1 × 10^5^) were seeded onto coverslips in a 6-well plate. Then, 3HP-β-LG (0.5 μg/ml) was added for 12 h. Then, the supernatant was replaced with DMEM containing 2% FBS. After 24 days, the cells were fixed by 4% paraformaldehyde (PFA; Sigma-Aldrich, St. Louis, MO), perforated by 0.2% Triton X-100, and blocked with 3% BSA (Amresco, LLC, Solon, OH). Next, cells were incubated overnight with anti-3HP-β-LG mAb or anti-β-actin mAb (1:1,000) at 4°C. After five washes, the cells were incubated with Alexa Fluor 488-labeled donkey anti-mouse IgG (1, 1,000, Thermo Fisher Scientific, Wilmington, DE, USA) at room temperature for 1h. After five washes, the coverslips were sealed with Prolong Gold Antifade reagent with 4,6-diamidino-2-phenylindole (Thermo Fisher Scientific) and scanned with the Leica SP8 confocal microscope.

## Results

### 3-Hydroxyphthalic Anhydride Modified Bovine Beta-Lactoglobulin Is the Most Potent Anhydride-Modified Protein Against Human Papillomavirus Entry Into the Target Cell

**Figure 1:**
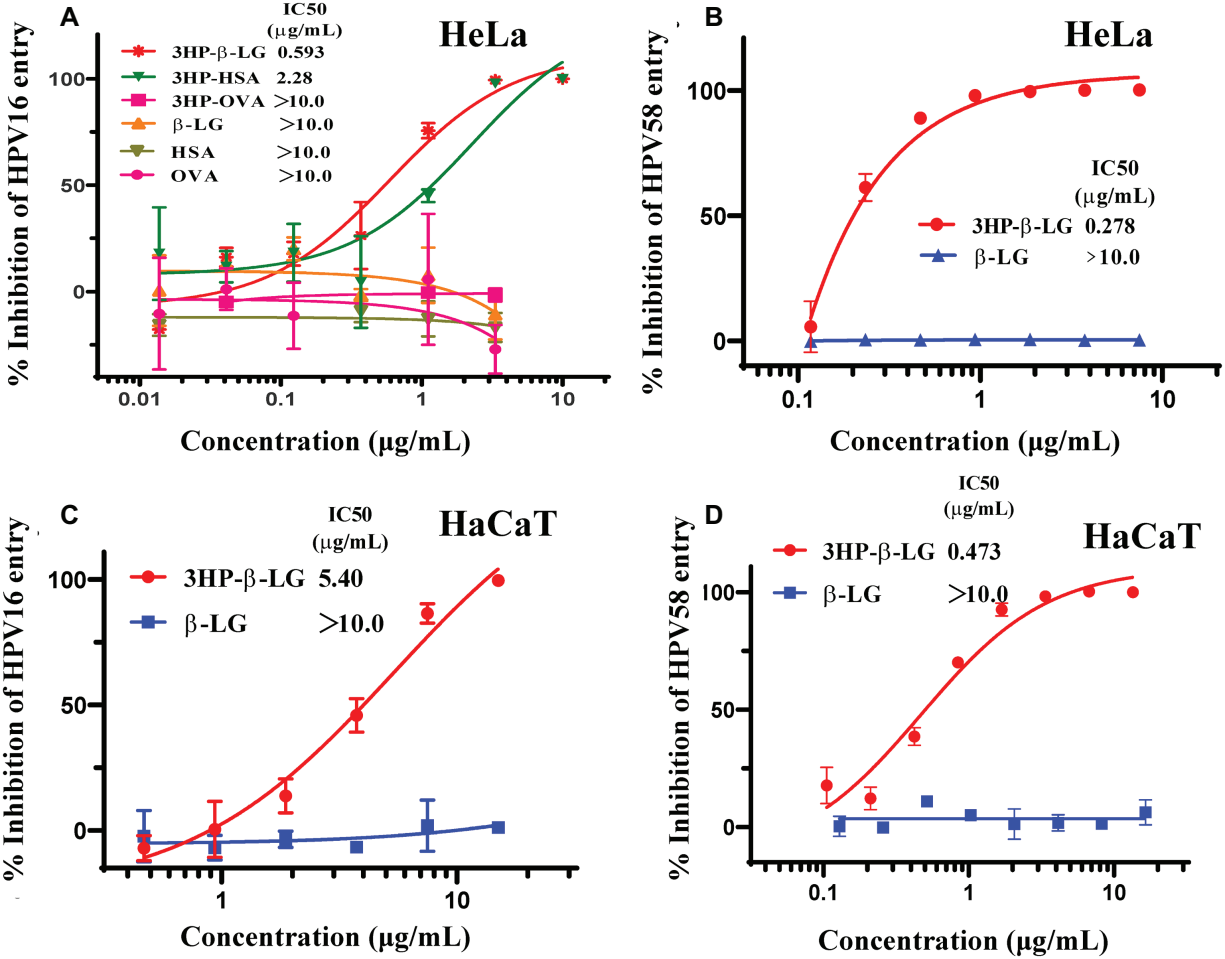
Anti-HPV activities of anhydride-modified proteins. **(A)** Inhibition of anhydride-modified proteins on entry of HPV16 PsV into HeLa cells. Protein candidates include human serum albumin (HSA), β-lactoglobulin (β-LG), and chicken ovalbumin (OVA). **(B)** Inhibition of 3HP-β-LG on entry of HPV58 PsV into HeLa cells. **(C)** Inhibition of 3HP-β-LG on entry of HPV16 PsV into HaCaT cells. **(D)** Inhibition of 3HP-β-LG on entry of HPV58 PsV into HaCaT cells. Each sample was tested in triplicate, and the experiment was repeated twice. Data from a representative experiment are presented as mean ± standard deviation (SD).

Our previous studies have shown that 3-hydroxyphthalic anhydride-modified bovine beta-lactoglobulin (3HP-β-LG) is a promising HPV entry inhibitor against entry of PsV of both high-risk and low-risk HPV types (Lu et al., 2013). This inhibitory activity was attributed to the interaction of the increased net negative charges on β-LG after 3-hydroxyphthalic anhydride modification (Lu et al., 2013). One may ask whether all proteins with increased net negative charges can inhibit HPV entry into the target cell. To answer this question, we compared the inhibitory activity of 3HP-β-LG with that of 3-hydroxyphthalic anhydride-modified human serum albumin (3HP-HSA) and 3-hydroxyphthalic anhydride-modified chicken ovalbumin (3HP-OVA) against entry of HPV16 PsV into HeLa cells, using the unmodified β-LG, HSA, and OVA as controls. As shown in Figure 1A, 3HP-β-LG exhibited potent anti-HPV activity in a dose-dependent manner with an IC_50_ (the half maximal inhibitory concentration) of 0.59 μg/ml, which is more potent than that of 3HP-HSA (IC_50_: 2.28 μg/ml). 3HP-OVA and all three unmodified proteins at the concentration as high as 10 μg/ml showed no significant inhibitory activity against HPV16 PsV entry into the target cell. These results confirm that not all, just some, proteins, such as β-LG and HSA, modified by 3-hydroxyphthalic anhydride gain the ability to inhibit HPV infection.

In our previous studies, we proved the antiviral activity of 3HP-β-LG against entry of the high-risk HPV types 16 and 18 and low-risk HPV type 6 into the target cell (Lu et al., 2013). Aside from these three representative HPV types worldwide, another important high-risk type, HPV58, is found with high incidence in China. To determine whether 3HP-β-LG is also effective against HPV58, we constructed the HPV58 pseudovirus and tested its sensitivity to 3HP-β-LG (control: β-LG). As shown in Figure 1B, 3HP-β-LG could also inhibit HPV58 PsV entry into the target cell in a dose-dependent manner (IC_50_ of 0.28 μg/ml), confirming that 3HP-β-LG has broad-spectrum anti-HPV activity.

Considering that the HaCaT cells express abundant integrin and heparan sulfate proteoglycan (HSPG), the receptors for HPV (Aksoy et al., 2014; Kumar et al., 2014) and act as target cells for HPV infection, we also evaluated the inhibitory activity of 3HP-β-LG against HPV16 and HPV58 PsV entry into HaCaT cells. As shown in Figures 1C,D, 3HP-β-LG was also effective against entry of HPV16 and HPV58 PsV into HaCaT cells, suggesting that 3HP-β-LG acts on the virus, rather than the target cell.

### 3-Hydroxyphthalic Anhydride Modified Bovine Beta-Lactoglobulin Binds to the L1 Protein and the Peptides Derived From the Positively Charged Residue-Enriched Region in Human Papillomavirus L1 Protein

**Figure 2:**
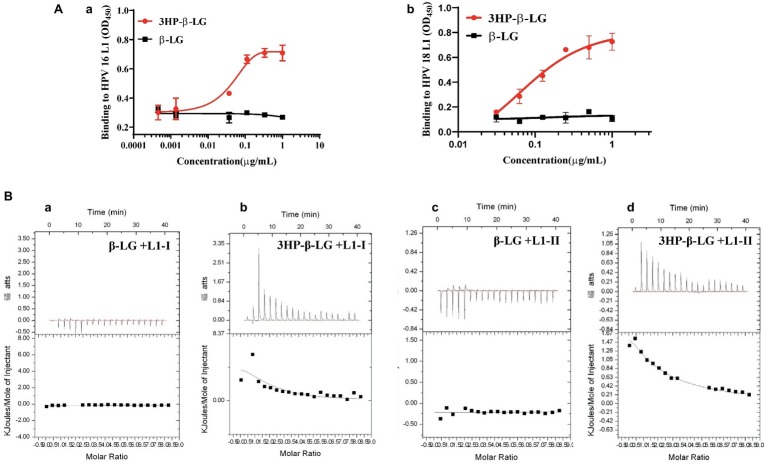
Interaction of 3HP-β-LG with HPV L1 protein and with peptides derived from the positively charged region in the HPV L1 protein. **(A)** Binding of 3HP-β-LG (control: β-LG) with L1 protein of HPV16 **(a)** and HPV18 **(b)** in ELISA. Each sample was tested in triplicate, and the experiment was repeated twice. Data from a representative experiment are presented as mean ± SD. **(B)** Binding of 3HP-β-LG (control: β-LG) with L1–1 peptide **(a** and **b)** or L1-II peptide **(c** and **d)** derived from the region of residues 474–488 (LKAKPKFTLGKRKAT) or region of residues 493–505 (STSTTAKRKKRKL) of HPV16, respectively, in ITC.

As we proposed before, 3HP-β-LG inhibited HPV PsV entry into the target cell possibly through the binding of the negatively charged residues in 3HP-β-LG with the positively charged residues in a protein on the surface of the viral particle, thereby blocking the interaction between viral protein and receptor on the target cell (Lu et al., 2013). However, it is still unclear which protein on the virus surface may be involved in this interaction. The most abundant protein on the HPV particle is L1 protein. Here we detected the interaction between 3HP-β-LG and HPV L1 protein by ELISA. As shown in Figures 2Aa,Ab, 3HP-β-LG could strongly bind to the L1 protein of both HPV16 and HPV18, while the unmodified β-LG protein could not.

The C-terminal positively charged region of HPV L1 was proved to regulate the binding of HPV to the receptor (Sibbet et al., 2000; Bousarghin et al., 2003). This region was exposed on the viral surface, and involved in the binding to cell surface receptors or neutralizing antibodies. Therefore, we synthesized two positively charged peptides in this region of L1 protein, L1-I (residues 474–488) and L1-II (residues 493–505). The binding ability of 3HP-β-LG and β-LG to these peptides was measured by isothermal titration calorimetry (ITC). As shown in Figure 2B, β-LG had no obvious binding to L1-I or L1-II peptide, while 3HP-β-LG showed high binding ability against both L1-I (binding constant K_α_ value equals 2.31 × 10^5^/M) and L1-II (binding constant *K*_α_ value equals 1.04 × 10^5^/M). These results suggested that 3HP-β-LG may bind to the positively charged sites in the C-terminal region of L1 protein on the HPV surface to block the interaction between the viral particle and cell receptor.

### 3-Hydroxyphthalic Anhydride Modified Bovine Beta-Lactoglobulin Did Not Inactivate Human Papillomavirus Pseudovirus, but, Instead, Blocked Entry of Human Papillomavirus Pseudovirus Into the Target Cell *via* Its Inaction With Virus, Not Cells

**Figure 3:**
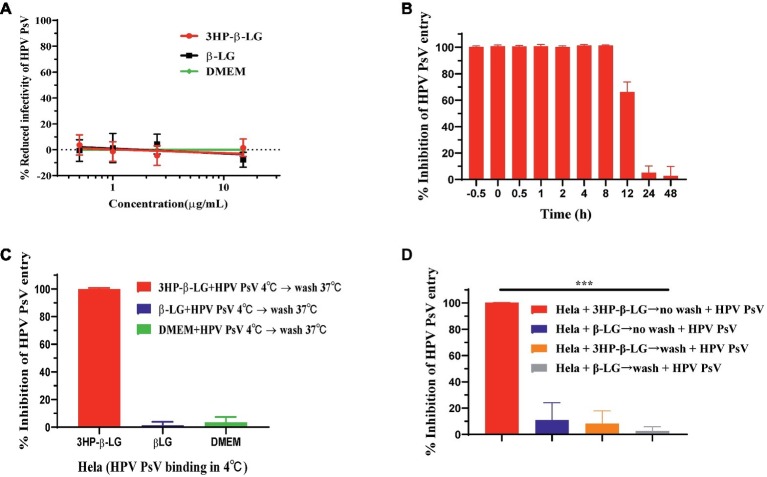
3HP-β-LG inhibited HPV PsV entry by targeting virus, not cells. **(A)** 3HP-β-LG was unable to inactivate HPV PsV. HPV PsV was incubated with 3HP-β-LG (control: β-LG) at room temperature for 2 h and then separated by PEG-8000 to analyze its entry activity. The DMEM-treated virus was taken as negative control. **(B)** 3HP-β-LG blocked HPV PsV entry into the target cell. HeLa cells were incubated with HPV PsV, while 3HP-β-LG (15 μg/ml) was added at different time points (−0.5, 0, 0.5, 1, 2, 4, 8, 12, 24, and 48 h) to analyze the inhibitory activity. **(C)** 3HP-β-LG blocked HPV PsV entry by targeting virus. Temperature shift assay for anti-HPV activity of 3HP-β-LG (control: β-LG). Diluted 3HP-β-LG or β-LG at 15 μg/ml was mixed with HPV PsV and prechilled at 4°C for 30 min; then they were added to HeLa cells for 12 h. After washing twice, the cells were further incubated at 37°C for 72 h and then analyzed for fluorescent integrated density. **(D)** 3HP-β-LG inhibited HPV PsV entry by targeting the virus, not the host cell. HeLa cells were incubated with 3HP-β-LG (control: β-LG) at 37°C for 1 h with/without washing by DMEM before addition of HPV PsV. Each sample was tested in triplicate, and the experiment was repeated twice. Data from a representative experiment are presented as mean ± SD. Asterisks represent significant differences. ^***^p < 0.001.

To determine whether 3HP-β-LG could inactivate HPV PsV, we incubated HPV PsV with 3HP-β-LG at room temperature for 2 h and then removed 3HP-β-LG by PEG-8000 precipitation. As shown in Figure 3A, the treated virus could still infect the target host cell with no reduced activity, compared the control virus (DMEM- or β-LG-treated), suggesting that the binding between 3HP-β-LG and HPV PsV may be noncovalent and reversible so that 3HP-β-LG cannot permanently inactive the virus.

HPV entry into the host cell takes more than 10 h, a relatively slow process compared to other viruses (Aksoy et al., 2017), which provides a long window period for an entry inhibitor to bind with the viral protein responsible for interaction with the receptor on the target cell and block viral entry. Here, we performed a time-addition assay to pinpoint the window of functional 3HP-β-LG blocking of HPV entry into the target cell. As shown in Figure 3B, 3HP-β-LG (15 μg/ml) could fully inhibit HPV16 PsV entry when it was added to the cells 0.5 h before and 0.5, 1, 2, 4, and 8 h after addition of HPV16 PsV, respectively, while about 70, 10, and 5% of HPV16 PsV entry were blocked when 3HP-β-LG was added to the cells 12, 24, and 48 h after addition of HPV16 PsV, respectively (Figure 3B). This result suggests that during this relatively long period of entry process (Aksoy et al., 2017), the entry inhibitor 3HP-β-LG remains sufficiently active to inhibit HPV PsV entry into the target cell.

To confirm the functional stage of 3HP-β-LG in blocking viral entry, we used a temperature shift assay to slow down the viral infection process. At 4°C, HPV PsV could bind to cell receptors, but could not fully enter the cytoplasm because the speeds of membrane fusion and cytoskeletal transformation are largely reduced at such low temperature. We incubated HPV PsV and HeLa cells at 4°C for 12 h so that HPV could bind to the cell surface. Then we washed away the unbound HPV before shifting the cells to 37°C for further 72 h of incubation. When 3HP-β-LG was added into the cells at the beginning and then removed by washing, no viral entry occurred (Figure 3C). This proved that 3HP-β-LG blocked the attachment between PsV and cell receptor at the earliest stage of infection. To determine whether 3HP-β-LG could also bind to the receptor on the target cell surface to block HPV entry, we incubated HeLa cells with 3HP-β-LG (control: β-LG) at 37°C for 1 h. The cells were washed with DMEM to remove the unbound proteins, or not washed (as control), before addition of HPV PsV. Under the washing conditions, HPV PsV entry into the HeLa cells was not blocked, while under the non-washing conditions, HPV PsV entry into the HeLa cells was effectively blocked. No inhibition was observed in the β-LG control groups, no matter whether the β-LG-treated HeLa cells were washed or not washed (Figure 3D). These results suggest that 3HP-β-LG inhibition of HPV PsV entry into HeLa cells does not result from its binding to HPV’s receptor on the host cell.

### 3-Hydroxyphthalic Anhydride Modified Bovine Beta-Lactoglobulin Could Not Penetrate Into the Cell and the Vaginally Applied 3-Hydroxyphthalic Anhydride Modified Bovine Beta-Lactoglobulin Did Not Enter Into the Blood Circulation

**Figure 4:**
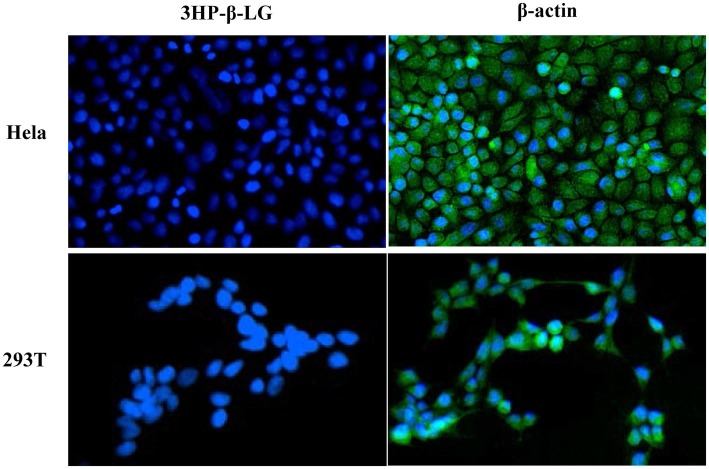
3HP-β-LG could not enter into the target cell. After incubation with HeLa and 293 T cells, 3HP-β-LG in the cells was detected by immunofluorescence assay using anti-3HP-β-LG mAb (green). β-actin was used as positive control. Cell nuclei were stained by 4,6-diamidino-2-phenylindole (blue).

To determine whether intravaginally applied 3HP-β-LG could enter into epithelial cells, we incubated HeLa cells and 293 T cells with 3HP-β-LG for 24 h and then washed the cells three times using PBS. The cells were stained with mouse anti-3HP-β-LG antibody or anti-β-actin antibody, as a control, and Alexa Fluor 488-labeled donkey anti-mouse IgG. Different from the anti-β-actin antibody-treated cells that showed strong fluorescence signals, anti-3HP-β-LG antibody-treated cells displayed no significant fluorescence signals inside or outside the cells (Figure 4). These results indicate that 3HP-β-LG could neither enter into the cell nor bind the cell surface proteins, including the receptor(s) for HPV.

**Figure 5:**
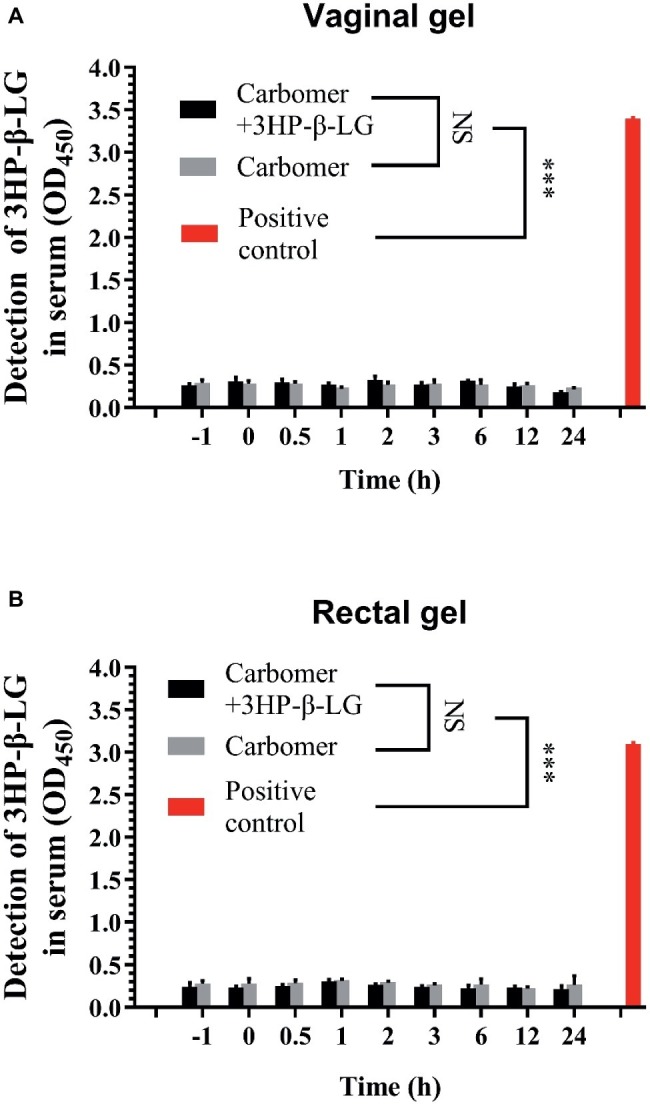
3HP-β-LG could not enter into blood circulation when applied through the vagina or anus. **(A)** Concentration of 3HP-β-LG in sera of monkeys through vaginal administration. **(B)** Concentration of 3HP-β-LG in sera of monkeys through rectal administration. 3HP-β-LG was formulated in a carbomer gel (1.8 mg/each) and administered through the vagina or anus of rhesus macaques. Their blood samples were collected at different time points before and after the application (−1 to 24 h). 3HP-β-LG in the blood samples was analyzed by ELISA. Each sample was tested in triplicate, and the experiment was repeated twice. Data from a representative experiment are presented as mean ± SD. The asterisks represent significant differences: ^*^p < 0.05; ^**^p < 0.01; ^***^p < 0.001.

To investigate whether the topically applied 3HP-β-LG enters the blood circulation, a carbomer gel with or without 3HP-β-LG was topically administered in the vagina or anus of rhesus macaques. Their blood samples were collected at different time points before and after gel application. The concentration of 3HP-β-LG in the blood samples and that in the gel (as positive control) were quantified with ELISA using anti-3HP-β-LG mAb. As shown in Figure 5, no 3HP-β-LG was detected in the blood samples of rhesus macaques vaginally or rectally administered with carbomer gel with or without 3HP-β-LG, confirming that the topically applied 3HP-β-LG does not enter the blood circulation.

## Discussion

About 10–20% of acute HPV infections become persistent, leading to various forms of cancer (Shanmugasundaram and You, 2017), such as the cervical cancer. Although several multivalent prophylactic HPV vaccines have been used in clinics to prevent HPV infection and cervical cancer development, they have no effect against pre-existing HPV infection in middle-aged and elderly women, the population at high risk for cervical cancer. Therefore, it is essential to develop effective and safe therapeutic agents for treatment of HPV infection in order to reduce the morbidity of cervical cancer.

Viral entry inhibitors have been proven effective and safe for the treatment of viral infections (Jiang et al., 1993; Dando and Perry, 2003; Lu et al., 2016; Li et al., 2017; Su et al., 2019). We have previously reported that an HPV entry inhibitor, 3HP-β-LG, is highly effective in blocking the entry of HPV, both high- and low-risk types, into the host cell (Lu et al., 2013). In a randomized clinical trial, topical application of the vaginal gel containing 3HP-β-LG has shown excellent efficacy and safety to treat high-risk HPV infections (Guo et al., 2016a,b). However, how 3HP-β-LG inhibits HPV entry into the target cell is still unclear.

**Figure 6:**
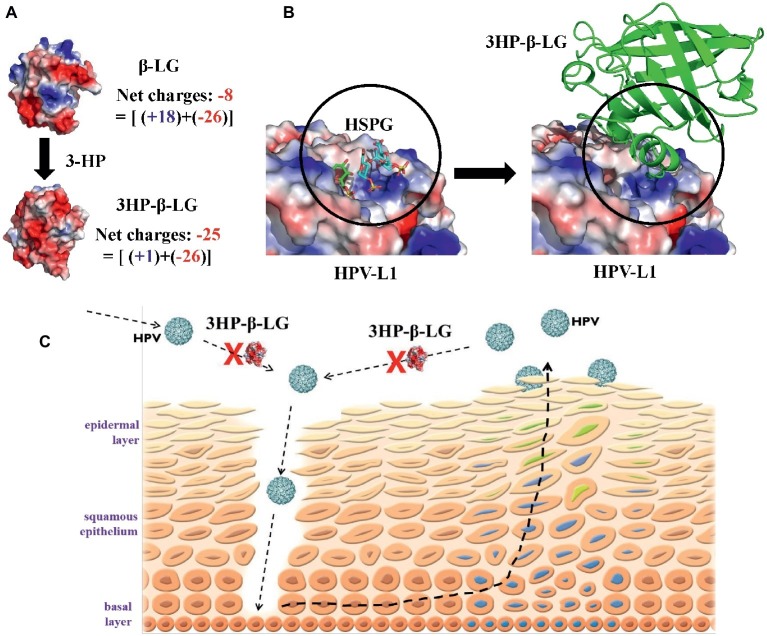
Proposed mechanism by which 3HP-β-LG inhibits HPV infection in cervical mucosa. **(A)** The increased net negative charges on β-lactoglobulin after modification of the protein with 3-hydroxyphthalic anhydride. The structure of 3HP-β-LG was modeled by COOT (Emsley et al., 2010) using the crystal structure of β-LG (PDB entry: 1BEB). The structures were shown as an electrostatic surface by Pymol (Alexander et al., 2011). **(B)** Predicted interactions between HPV L1 pentamer and 3HP-β-LG. Crystal structure of HSPG-bound HPV L1 pentamer (PDB entry: 5W1O) was shown as electrostatic surface (Dasgupta et al., 2011), and the docking structure between HPV L1 and 3HP-β-LG was generated by AutoDock (Trott and Olson, 2010). **(C)** Schematic illustration showing how 3HP-β-LG inhibits HPV entry and infection. The newly invaded or newly released HPV particles access the basal layer through the mucosal lesion, or breaks, and bind to the HSPG receptor on the basement membrane, resulting in the entry of HPV into and replication in mucosal epithelial cells. 3HP-β-LG can bind with HPV L1 protein, through the electrostatic interaction between the negatively charged region in 3HP-β-LG and the positively charged region in L1 protein, to block the attachment of HPV to the HSPG receptor, resulting in inhibition of HPV infection in vaginal mucosa.

In our previous study, we demonstrated that the inhibitory activity of 3HP-β-LG on HPV PsV entry into the target cell is closely correlated with the number of the positively charged lysine and arginine residues in 3HP-β-LG, as modified, *i.e.*, the net negative charges on 3HP-β-LG (Lu et al., 2013), suggesting that the negatively charged residues on 3HP-β-LG play an important role in 3HP-β-LG-mediated inhibition of HPV infection. As shown in Figure 6A, the unmodified bovine β-LG contains 18 positively charged residues (15 Lys and 3 Arg) and 26 negatively charged residues (16 Asp and 10 Glu) (Qin et al., 1999), thus having a net charge of −8. In 3HP-β-LG, 14 out of the 15 Lys and all 3 Arg were modified by 3HP at 6 mM (Lu et al., 2013), resulting in the neutralization of 17 of the 18 positive charges. Therefore, the net charge of 3HP-β-LG becomes −25, making 3HP-β-LG be active in binding to a viral protein with high positive net charges and inhibiting virus infection.

In this study, we demonstrated that 3HP-β-LG could strongly bind to the L1 protein on the HPV16 PsV, while the unmodified β-LG protein could not (Figure 2A). Using ITC analysis, we showed that unlike β-LG, 3HP-β-LG could strongly interact with the positively charged residue-enriched C-terminal regions (residues 474–488 and 493–505) of L1 protein, which is responsible for the attachment of the HPV particle to the host cell, possibly through its interaction with the cellular receptor(s).

One may argue that 3HP-β-LG may bind to any virus with high net positive charges on its surface protein and inhibit its infection. Indeed, 3HP-β-LG can also inhibit infection of simian immunodeficiency virus (SIV) (Wyand et al., 1999) and HSV (Neurath et al., 1996), but it is not effective against infection of MERS-CoV and VSV (unpublished data). In this study, we showed that 3HP-modified HSA (3HP-HSA) was also effective, while 3HP-modified OVA (3HP-OVA) was not effective in inhibiting HPV PsV entry (Figure 1), even though 3HP-OVA also contains high net negative charges on its surface. Thus, aside from having high net negative charges, these results suggest that the active site in 3HP-β-LG for binding to the viral protein must possess a conformation that fits with that in the corresponding site in the viral protein (Figure 6B), enabling 3HP-β-LG to strongly and closely interact with the viral protein to block HPV entry into the host cell. Indeed, the molecular docking analysis indicated that 3HP-β-LG could strongly bind to the positively charged region of HPV L1 protein, which is the binding site for the negatively charged HSPG receptor for HPV (Figure 6B). 3HP-β-LG has many more negative charges on the surface, and the interface between 3HP-β-LG and HPV-L1 is much larger than the HSPG molecule. Therefore, 3HP-β-LG can competitively bind to HPV L1 to block the binding of the HSPG receptor.

Based on the results obtained from this study, we proposed a mechanistic model of 3HP-β-LG for inhibiting HPV infection in the cervical mucosa (Figure 6C). The newly invaded HPV accesses the basal cell layer through micro lesions or breaks in the cervical epithelium and binds to the HSPG receptor on the basement membrane through interaction between the positively charged region in L1 protein of HPV and the negatively charged region in HSPG. This interaction results in a series of conformational changes in the capsid, which leads to protease digestion of L2 and exposure of its N terminus, thus initiating entry of HPV into to the epithelial cells (Kines et al., 2009; Pyeon et al., 2009). After HPV infects the epithelial cell, it gradually matures along with the host cell cycles and moves toward the top layer of epithelia, followed by eventual release from the epidermal cells. The newly released HPV particles access the basement membrane through micro lesions of the cervical epithelium and start another infection and replication cycle, gradually establishing persistent infection and promoting the development of cervical cancer.

3HP-β-LG that is topically applied in the vagina can bind to the L1 protein of the newly invaded or the newly released HPV and block the attachment of HPV to the basement membrane, thereby inhibiting the entry of HPV into the host cells for replication. After treatment with 3HP-β-LG for a certain period of time (e.g., 2 months), the newly produced HPV-free epithelial cells in the lower part of the mucosal epithelium are expected to move upward and push the HPV-infected cells away from the vaginal mucosa (this movement is accelerated during the menstrual period), making all layers of mucosal epithelium free of HPV infection.

Considering that the presence of micro lesions or breaks in the cervical epithelium, possibly caused by sexual activity, is the requirement for HPV to access the HSPG receptor on basement membrane (Bousarghin et al., 2003), combinational use of some biocompatible materials for mucosa wound healing, such as human collagen proteins (Hua et al., 2019), may have synergistic or complementary effects against HPV infection.

The results from the time-of-addition assay, cell washout assay, and virus inactivation indicate that 3HP-β-LG could interact with the virions, rather than the cells, to effectively block the entry of HPV PsV into the target cell, but could not inactivate the virions (Figure 3). These biological properties of 3HP-β-LG make it an ideal anti-HPV agent for topical application to treat HPV infection because it can interact with HPV particles on the vaginal mucosa and block the virions binding to the receptor, e.g., HSPG, on basement membrane. Since 3HP-β-LG cannot enter into the cell or the blood circulation, it is not expected to cause systemic toxicity to human or induce harmful 3HP-β-LG-specific antibodies. Therefore, the topical formulations containing 3HP-β-LG can be safely used to treat local HPV infection.

## Data Availability Statement

All datasets generated for this study are included in the manuscript/supplementary files.

## Ethics Statement

The animal study was reviewed and approved by Institute of Laboratory Animal Science, Chinese Academy of Medical Sciences and Peking Union Medical College (approval number: ILAS-VL-2015-001).

## Author Contributions

SJ, LSu, and YZ conceived and designed the experiments. CH, CW, LSi, and QW conducted the experiments. YZ performed the computer modeling and analysis of the interaction between 3HP-β-LG and HPV L1 protein. CH, YZ, LSu, and SJ analyzed the data and wrote the manuscript. All authors have read and approved the final manuscript.

### Conflict of Interest

The authors declare that the research was conducted in the absence of any commercial or financial relationships that could be construed as a potential conflict of interest.

## Footnotes

^1^http://home.ccr.cancer.gov/lco/default.asp
